# Characterization of Graphite Oxide and Reduced Graphene Oxide Obtained from Different Graphite Precursors and Oxidized by Different Methods Using Raman Spectroscopy

**DOI:** 10.3390/ma11071050

**Published:** 2018-06-21

**Authors:** Roksana Muzyka, Sabina Drewniak, Tadeusz Pustelny, Maciej Chrubasik, Grażyna Gryglewicz

**Affiliations:** 1 Institute for Chemical Processing of Coal, 1 Zamkowa St., 41-803 Zabrze, Poland; rmuzyka@ichpw.pl (R.M.); mchrubasik@ichpw.pl (M.C.); 2 Department of Optoelectronics, Silesian University of Technology, 2 Akademicka St., 44-100 Gliwice, Poland; tadeusz.pustelny@polsl.pl; 3 Department of Polymer and Carbonaceous Materials, Wroclaw University of Technology, 4/9 Gdańska St., 50-344 Wroclaw, Poland; grazyna.gryglewicz@pwr.edu.pl

**Keywords:** graphite, graphite oxide, reduced graphene oxide, modified Hummers method, oxidation, reduction, Raman spectroscopy

## Abstract

In this paper, the influences of the graphite precursor and the oxidation method on the resulting reduced graphene oxide (especially its composition and morphology) are shown. Three types of graphite were used to prepare samples for analysis, and each of the precursors was oxidized by two different methods (all samples were reduced by the same method of thermal reduction). Each obtained graphite oxide and reduced graphene oxide was analysed by X-ray diffraction (XRD), X-ray photoelectron spectroscopy (XPS) and Raman spectroscopy (RS).

## 1. Introduction

Carbon-based materials are very interesting due to their stable physicochemical properties and low cost, as well as other characteristics [1]. These materials have been utilised in many applications, e.g., supercapacitors and thin film transistors [1,2,3]. One of the most interesting types of these carbon-based materials is reduced graphene oxide (rGO). This material can be used for many purposes e.g., in gas sensing structures [2,4,5]. However, the properties of the gas sensors depend on the properties of sensor layers [6], which are related to the preparation method of the material.

rGO is obtained by the oxidation of graphite (the obtained material is called graphite oxide (GRO)) followed by reduction and exfoliation [4,7]. Currently, one of the most popular methods of obtaining graphene oxide is the method proposed by Hummers [8,9,10,11], but other methods (e.g., that proposed by Staudenmeier and Brodie) are also well-known [8,10]. The reduction step can also be completed through several methods, such as the environmentally methods of flash photoreduction, hydrothermal, catalytic reduction and thermal annealing [7,12,13,14].

Based on the literature [5,7,8,15,16,17] the kind of graphite and the oxidation/reduction method have large impacts on the kind of functional groups present in the final product (in rGO and GRO). Materials prepared through different methods will have difference properties. For this reason, it is necessary to characterize products from different preparation methods. The characterization of carbon materials can be performed using many methods, but one of the more interesting methods is Raman spectroscopy (RS). This method gives a large amount of information about the testing materials and, importantly, is a nondestructive method [18,19]. The typical Raman spectrum of carbon materials contains bands marked as D, G and 2D [20]. The D band (located near 1350 cm^−1^) results from the presence of vacancies or dislocations in the graphene layer and at the edge of this layer. This band is also related to the presence of defects in the material [20,21,22]. The next band, the G peak, is related to the in-plane vibration of sp^2^ hybridized carbon atoms and is located near 1580 cm^−1^ [21,23]. The last peak (2D) is related to the number of graphene layers. This band is sometimes also marked as G’ and is located near 2700 cm^−1^ [23,24]. Based on the data obtained from the Raman spectra, additional information about the carbon materials can be obtained by analysing the ratio of the intensities of the individual peaks. As an example, the I_D_/I_G_ ratio (intensity of the D peak to the intensity of the G peak) is related to the amount of defects present in the material [25,26], while the I_2D_/I_G_ ratio (intensity of the 2D peak to the intensity of the G peak) is related to the number of graphene layers in the material [27]; when the G band increases and the 2D band decreases, the number of layers in the material is assumed to increase [20].

The literature indicates that additional peaks may be visible in the Raman spectra of some graphene-based materials after deconvolution. In our previous work [28], we presented a method for deconvoluting Raman spectra. After deconvolution, a peak near 1620 cm^−1^ (marked as D’) and two (according to other papers- three) additional bands above 2440 cm^−1^ become visible. There are discrepancies in the name of these latter peaks, where the first peak above 2440 cm^−1^ is marked as D* or G* and the next peaks are marked as D+D’ or D+G and 2D’ or G+D’ [20,22,28]. Analysis of the intensity ratios of these peaks also gives additional information about the materials, e.g., an increase in the I_2D_/I_D+G_ (I_2D_/I_D+D’_) ratio indicates the restoration of the sp^2^ hybridized structure [22].

## 2. Materials and Methods

### 2.1. The Selection of the Graphites

**Figure 1:**
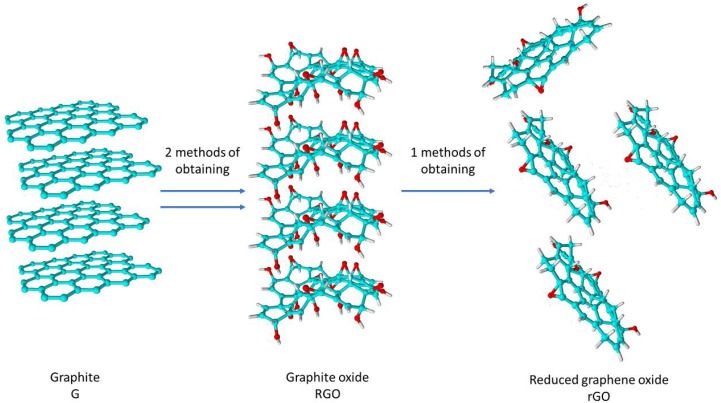
Scheme of the preparation of graphite oxide and reduced graphene oxide from graphite.

Two types of natural graphite, scale graphite (S) and flake graphite (F), and one synthetic graphite; electrode graphite (E), were used for the measurements. The contents of carbon in these analytical samples in the dry and ashless state are 99.2, 99.5 and 99.5, respectively. These graphites are characterized by a high degree of crystallinity and large crystallite dimensions; however, their properties differ between materials. Due to the spatial arrangement of the flake graphite structure, the bonds between neighbouring planes can be easily broken, resulting in significant anisotropy in this graphite. The natural scale graphite has a coarse crystal structure with large crystals. The synthetic graphite is characterized by a high purity and close similarity to natural graphite; however, it is obtained in a synthetic method from a properly prepared mass in an Acheson electric resistance furnace at a temperature of 2500–3000 °C [29]. The differences in the structural parameters of the examined graphites will be discussed in more detail in later sections of this paper. Nevertheless, these differences will affect the properties of the materials (graphite oxides and reduced graphene oxides) obtained from such graphites (a scheme of the preparation method and nomenclature is shown in Figure 1).

### 2.2. The Preparation of Graphite Oxide and Reduced Graphene Oxide

**Figure 2:**
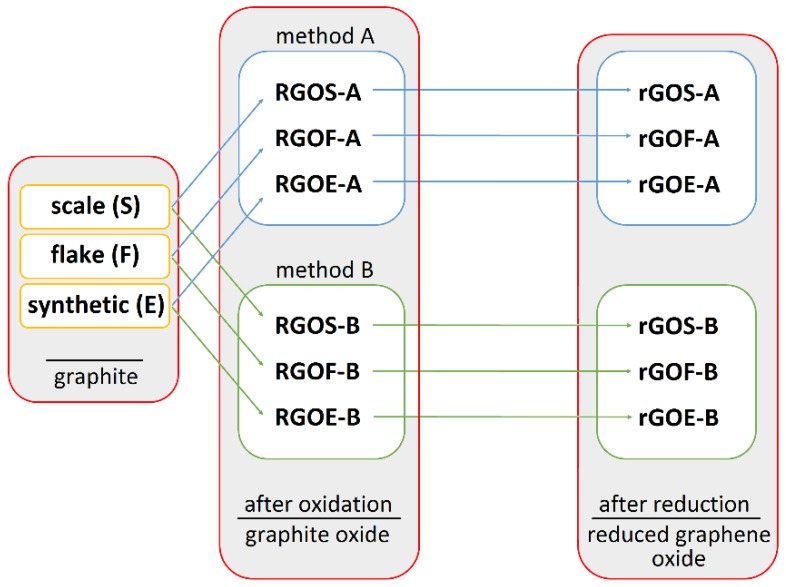
Diagram showing the abbreviations depending on the preparation step.

Graphite oxides were obtained from scale, flake and synthetic graphites by two methods denoted A and B (commercial graphite powders were ground in a planetary ball mill and sieved to a particle size < 20 µm and then oxidized according to the methods described in Table 1). Method A (Hummers’ method) is popular method which is used to obtain graphites oxides while method B is an original modification of Tour’s method. We will show (in this paper) that method B proved to be more effective in oxidation of graphites of various origins. The obtained products are denoted **GROS-A**, **GROF-A**, **GROE-A**, **GROS-B**, **GROF-B** and **GROE-B** according to the type of graphite used and the method of oxidation. The diagram showing used abbreviations (both for graphite, graphite oxide and reduced graphene oxide) is shown in Figure 2.

Reduced graphene oxides were obtained using high-temperature exfoliation/reduction according to the following process: the graphite oxide was blown under a flow of nitrogen for 30 min (5 mL/min) and subsequently annealed at 900 °C for 5 min. The obtained rGOs are denoted **rGOS-A**, **rGOF-A**, **rGOE-A**, **rGOS-B**, **rGOF-B** and **rGOE-B** according to the GRO used.

### 2.3. Characterization Methods of the Obtained Materials

#### 2.3.1. X-ray Diffraction (XRD)

X-ray diffraction measurements were conducted using an X’Pert PRO PW 3040/60 diffractometer (PANalytical, Quebec, Canada). The samples were deposited onto glass and analysed by using Cu Ka1 radiation with a voltage of 45 kV and a current of 30 mA.

#### 2.3.2. X-ray Photoelectron Microscopy (XPS)

XPS was performed using a PHI 500 VersaProbe spectrometer from ULVAC (Chigasaki, Japan) using an Al Kα anode radiation beam (1486.6 eV).

#### 2.3.3. Raman Spectroscopy (RS)

Spectroscopic studies were performed using an N-TEGRA Spectra platform (NT-MDT, Moscow, Russia). The vibrations of the molecules were excited using 532 nm wavelength. The exposure time was 10 s. Measurements were performed at ten different points of each sample.

## 3. Results

### 3.1. Results of the Graphite Measurements

The first step of the material characterization included determination of the crystallite diameter (L_a_), crystalline height (L_c_) and interplanar distance (d_002_).

As shown in Table 2, the analysed graphites are characterized by similar interplanar distances. Moreover, the crystallites have a large diameter (50–82 nm), which is desirable for subsequent synthetic processes. The crystalline height is from 20 to 29 nm, and the interplanar distance is in the range from 0.3362 to 0.3379 nm.

**Figure 3:**
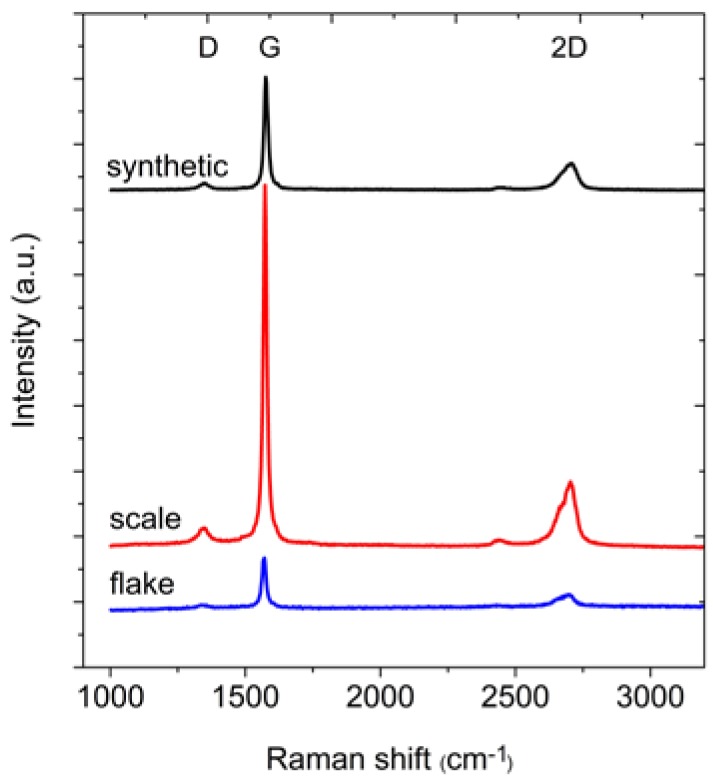
Raman spectra of the graphites.

In the next step of the measurements, the Raman spectra of the graphite samples were collected (Figure 3). From these spectra, the position and full width at half maximum (FWHM) of the D, G and 2D bands were determined (Table 3). The differences between the positions of the individual bands are small. The FWHM of the G peak is in the range 16–19 cm^−1^, and this small value indicates that the peak originates from the stretching vibrations of carbon bonds in the rings of the sp^2^ hybridized graphene layers. Meanwhile, the FWHM of the D band is in the range from 38 to 52 cm^−1^. This large FWHM means that the structure is perturbed, for example by the presence of heteroatoms or point dislocations in the structure (this band should not be observed in the case of perfect graphite [30]). However, the structures of all graphites are quite well-ordered, as confirmed by the A_G_/A_D_ ratio (where A represents the area under the peak). The area under the G peak is approximately 7–8 times larger than the area under the D peak. For all analysed graphites, the intensity of the G peak is much larger than the intensity of the D peak.

### 3.2. Results of the Graphite Oxide Measurements

The first stage of measurements of the graphite oxides included analysis using the XRD method. Based on the obtained data (Table 4), the largest interplanar distances (d_001_) are obtained for the GROFs. The smallest crystallite diameter, L_a_, is observed for the graphite oxides obtained from synthetic graphite, regardless of the oxidation method. The crystalline height (L_c_) is similar for all the GROs.

**Figure 4:**
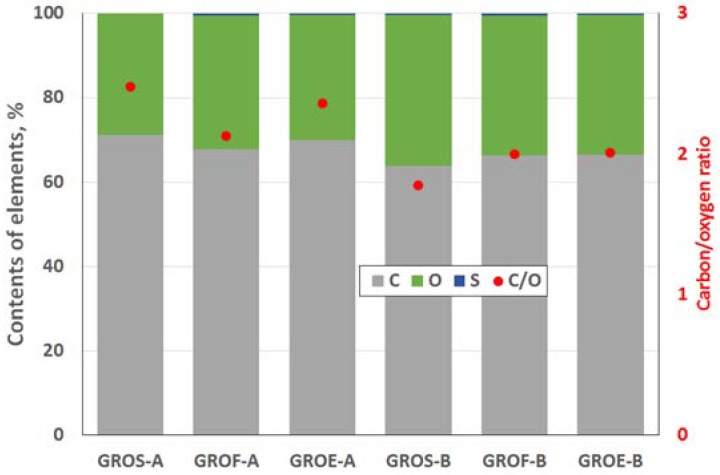
Elemental compositions of the graphite oxides determined by the XPS method, at %.

**Figure 5:**
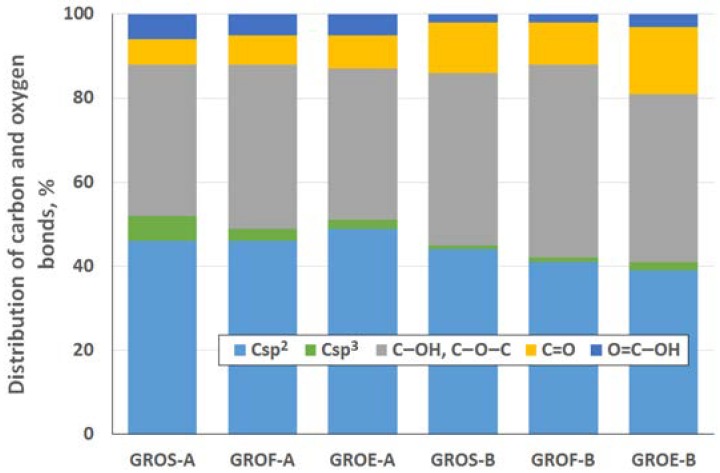
Distribution of carbon and oxygen bonds (in graphite oxide) determined by XPS, at %.

The GROs were also analysed using the XPS method (Figure 4 and Figure 5). All the graphite oxides are composed mainly of carbon and oxygen. The amount of carbon (>63%) in the GROs is much smaller than that in the graphites, regardless of the graphite type. The amount of oxygen is above 28%. The C/O ratio determined for the GROs oxidized by method A is larger than that for the GROs oxidized by method B. This result indicates that the B method was more effective for introducing oxygen into the graphite structure. The content of sulphur is low—below 0.5 at %.

To determine the distribution of carbon and oxygen bonds, the C 1s XPS spectra of the graphite oxides were deconvoluted into five components. After deconvolution, two peaks appear at energies (284.5 ± 0.1 eV and 285.4 ± 0.2 eV) that correspond to the sp^2^ and sp^3^ bonds of carbon. Moreover, peaks corresponding to carbon bonded to oxygen dominate the spectra of the GROs: three peaks (286.5 ± 0.3 eV, 287.6 ± 0.2 eV and 288.9 ± 0.3 eV) that correspond to hydroxyl/epoxy (C–OH, C–O–C), carbonyl/quinone (C=O) and carboxyl (O=C–OH) groups are identified. Based on the obtained data, the hydroxyl and epoxy groups are the most abundant. Smaller amounts of carbonyl and quinone groups are present, and the carboxyl group content is the smallest (more carbonyl and quinone groups and fewer carboxyl groups are present in the GROs oxidized by method B). Furthermore, the lowest degree of graphite oxidation is observed for the GROSs.

**Figure 6:**
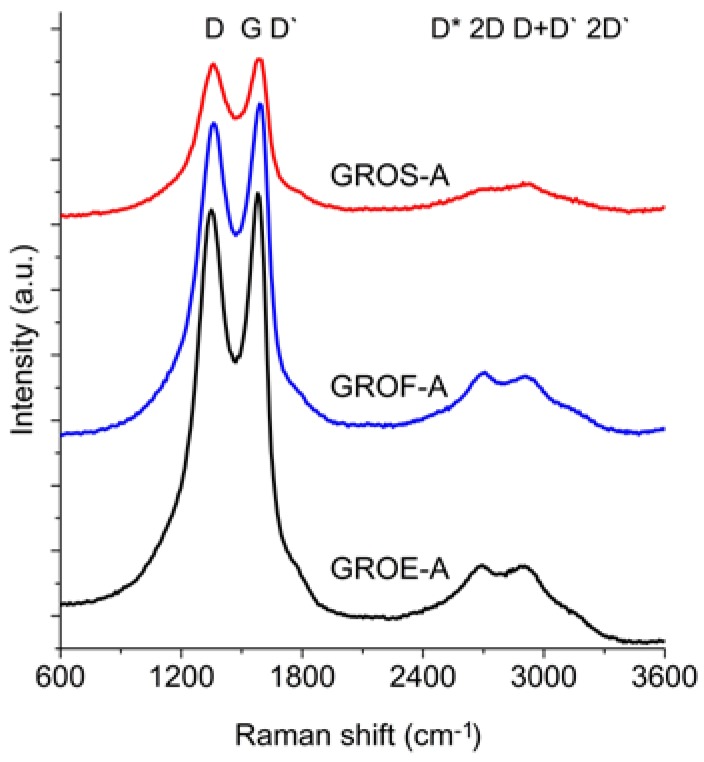
Raman spectra of graphite oxide (oxidation method A).

**Figure 7:**
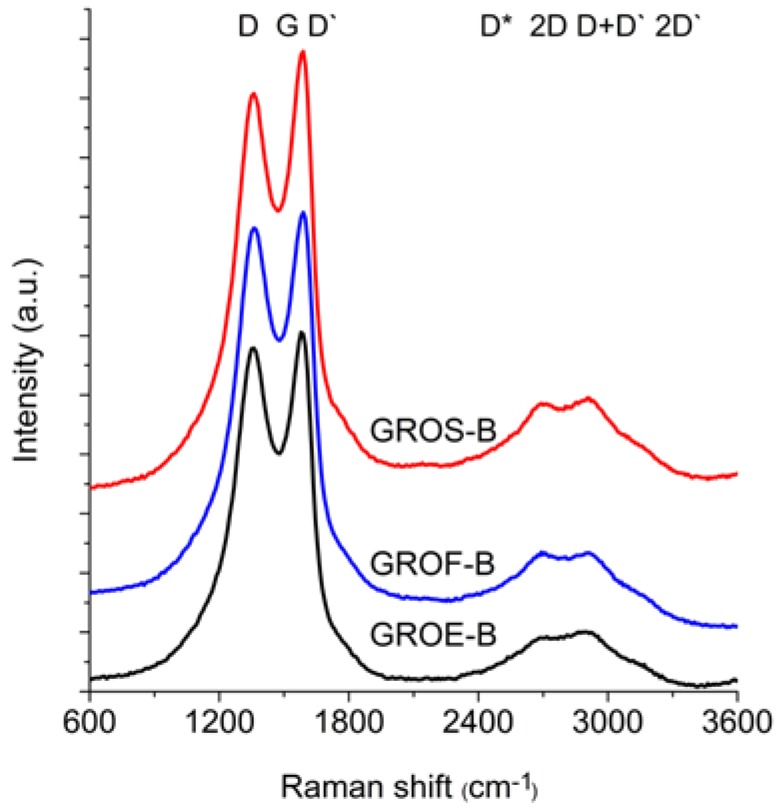
Raman spectra of graphite oxide (oxidation method B).

**Figure 8:**
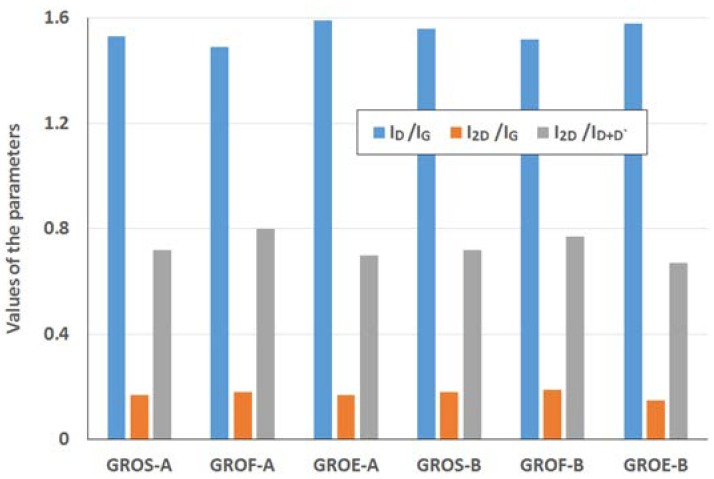
Average values of the parameters I_D_/I_G_, I_2D_/I_G_, and I_2D_/I_D+D’_ obtained from the Raman spectra of the graphite oxides.

All of the experiments whose results were described above (XRD and XPS) were performed to obtain information about the tested graphites and graphite oxides. This knowledge was helpful for interpreting the data obtained from Raman Spectroscopy (RS). The RS method enables much more detailed analysis of the changes related the introduction of oxygen atoms into the graphite structure, thus increasing the understanding of the unstructured phase. Figure 6 and Figure 7 show the Raman spectra of the GROs oxidized by methods A and B, respectively. All spectra were deconvoluted according to the method in reference [28] (for further analysis, the corresponding values were obtained from the deconvoluted spectra). The obtained data (FWHM and position of the peaks) are shown in Table 5, and the results of the calculations (I_D_/I_G_, I_2D_/I_G_, and I_2D_/I_D+D’_) are shown in Figure 8.

The spectra of GRO are characterized by intense D and G bands and a wide 2D band. Such an intense D band indicates the formation of defects in the structures. The increase in the intensity of the D band in relation to that of the G band indicates an increase in the amount of the disordered phase in the GROs. Unlike in the Raman spectra of the graphites, for the GROs, the D band is more intense than the G band, which is related to the formation of sp^3^ hybridized bonds as a result of the oxidation of graphite. Both the D and G bands of the GROs are wider than the D and G bands in the Raman spectra of graphite. Moreover, the G band of GRO is shifted towards lower wavenumbers, which confirms the presence of defects in the graphene layers.

The presence of oxygen atoms causes both an increase in the interplanar distance and changes in the characteristics of the vibrations in the material’s lattice. For this reason, the Raman spectra of oxidized graphites are characterized by more intense D peaks in comparison to the D peaks of the unoxidized graphites. If the degree of oxidation is larger, the intensity of this peak is also larger [20,31].

The FWHM values of the D and G bands are larger for the GROs than for the graphites. The wide G bands in the GROs are accompanied by a D’ band, the intensity of which is proportional to the number of defects. The FWHM of the D’ peak is in the range from 61 to 66 cm^−1^. A D’ band is observed in the spectra of graphene materials obtained from the oxidation or direct exfoliation of graphites and subsequent reduction of the GROs, which confirms the formation of structural defects [20,22,23,31,32]. Nevertheless, many researchers regard the wide G band in GROs as a single peak (not a sum of G and D’ peaks) [20].

The 2D band is less intense than the D band. Based on the position, shape and intensity of the 2D band obtained from deconvolution of the Raman spectrum into components (Lorentz curves), the number of graphene layers can be determined [33,34,35]. In the case of graphene monolayers, the 2D band occurs as a single peak near 2675 cm^−1^ [33]. In contrast, the 2D band of two graphene layers consists of four peaks: D*, 2D, D+D’ and 2D’. In this case, the D+D’ peak is more intense than the others. An increase in the number of layers reduces the intensities of the D* and 2D peaks and shifts the components towards higher wavenumbers.

In the spectra of the analysed GROs, four peaks: D* (2522–2558 cm^−1^), 2D (2695–2703 cm^−1^), D+D’ (2897–2910 cm^−1^) and 2D’ (3090–3118 cm^−1^), are obtained after deconvolution. The FWHM of the 2D peak is approximately 230 cm^−1^, which is much larger than that of the 2D peak of graphene (approximately 30 cm^−1^) [30]. The shape of the spectra and the observation of D*, 2D, D+D’ and 2D’ peaks indicate that the GROs consist of two or more graphene layers in an aggregate. Moreover, the higher intensity of the 2D band in relation to that of the G band confirms the presence of more defects in the structure. Please note that when the material is composed of two or more layers in an aggregate, the 2D band consists of two peaks, 2D_1_ and 2D_2_ [36] (marked according to the earlier designation as 2D and D+D’). In the case of the examined GROs, both the 2D and D+D’ peaks are observed.

The GROs obtained by method B are characterized by equal or lower I_2D_/I_D+D’_ ratios relative to those of the GROs prepared by method A. This difference suggests that more defects are present in the aggregates of the GROs prepared by method B. The largest I_D_/I_G_ ratio and the smallest I_2D_/I_G_ and I_2D_/I_D+D’_ ratios are observed for the oxides obtained from synthetic graphite, which may indicate that synthetic graphite is the most susceptible to oxidation and exfoliation.

### 3.3. Results of the Reduced Graphene Oxide Measurements

All reduced graphene oxides were examined by the same methods as those employed for the graphite oxides, and XRD measurements were performed first. The structural parameters of the rGOs are shown in Table 6. Following thermal exfoliation and reduction, the (001) band completely disappears from the diffraction patterns of the rGOs. Simultaneously, a (00X) band appears due to the partial removal of oxygen groups. Moreover, a more intense (002) is observed, which indicates the progressive reconstruction of the crystalline structure. The oxidation of graphite by method B can be concluded to lead to a larger separation of graphene layers (larger values of d_00X_ and d_002_) as a consequence of the greater degree of exfoliation that occurs during high-temperature processing. The values calculated on the basis of the 002 band are in the range (0.3373–0.3393 nm) for method A and in the range (0.3421–0.3440 nm) for method B. The largest values of the interplanar distances (d_00X_) are observed for the rGOF materials, while the smallest ones are observed for rGOE. The maintenance of a large distance between graphene layers may be related to the presence of significant amounts of oxygen in the rGOs and a large degree of defect formation in the graphene layers. The crystallite sizes of the rGOs differ depending on the type of graphite used and the oxidation method. The smallest values of L_c_ and L_a_ are obtained for rGOs fabricated from synthetic graphite. Moreover, analysis of the obtained parameters reveals that higher values of L_c_ and L_a_ are obtained for the rGO-A series independent of the type of graphite. This result suggests that a lower degree of exfoliation occurs for the GROs obtained by method A. The resulting rGOs (method A) are characterized by a lower susceptibility to high-temperature exfoliation (larger number of layers).

**Figure 9:**
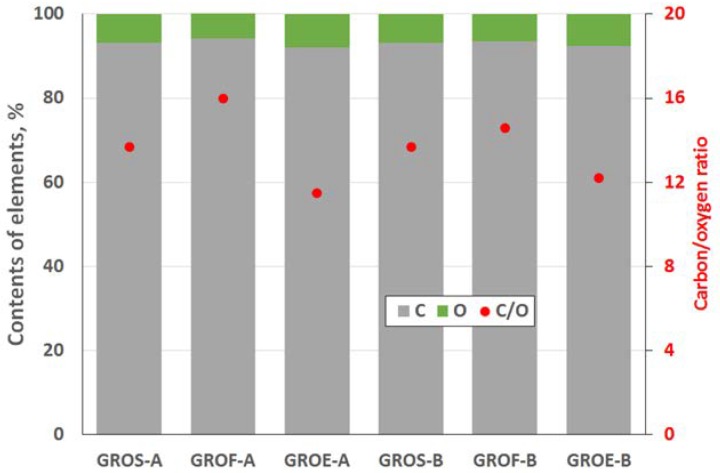
Surface elemental composition of the reduced graphene oxides determined by the XPS.

Based on the data obtained using the XPS method (Figure 9), a larger percentage of carbon and smaller percentage of oxygen are observed in the rGOs relative to those of the GROs, in which the amount of oxygen decreased 4–5 times. The degree of reduction of the rGO surface, represented by the C/O ratio, depends on the type of graphite used and increases in the order rGOE < rGOS < rGOF.

**Figure 10:**
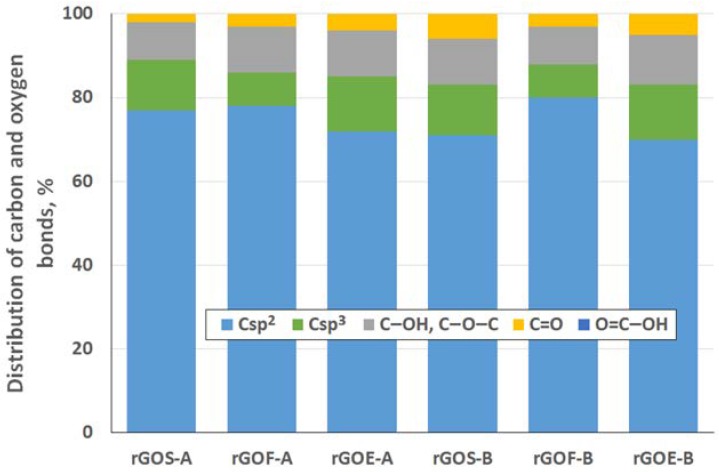
Distribution of carbon and oxygen bonds (reduced graphene oxide) determined by XPS, %.

As was done for the GROs, the distribution of carbon and oxygen bonds was determined for rGO using the XPS method. Analysis of the XPS C 1s spectra after deconvolution (data presented in Figure 10) confirms that the highest degree of reduction of the rGOs is obtained for the samples prepared from flake graphite. The highest percentage of sp^2^ hybridized carbon (78% for A method and 80% for B method) occurs for these samples, and their percentage of sp^3^ hybridized carbon is the smallest among all the rGOs. Meanwhile, the largest amount of sp^3^ hybridized carbon in the rGOs is obtained for the samples prepared from synthetic graphite. As a reminder, the lowest degree of reduction, expressed as the C/O ratio, also occurs in the rGOEs.

Deconvolution of the C 1s spectra shows that the rGOs mainly contain hydroxyl and epoxy groups. The carbon in the carbonyl and quinone groups accounts for 2% to 6% of the total content, and only a trace content of carboxyl carbons is observed. Following thermal exfoliation/reduction to produce rGO, the amounts of hydroxyl and epoxy groups decreases almost four times (Figure 10) compared to those in GRO (Figure 5). Furthermore, fewer carbonyl and quinone groups are present in the structures of the rGOs.

**Figure 11:**
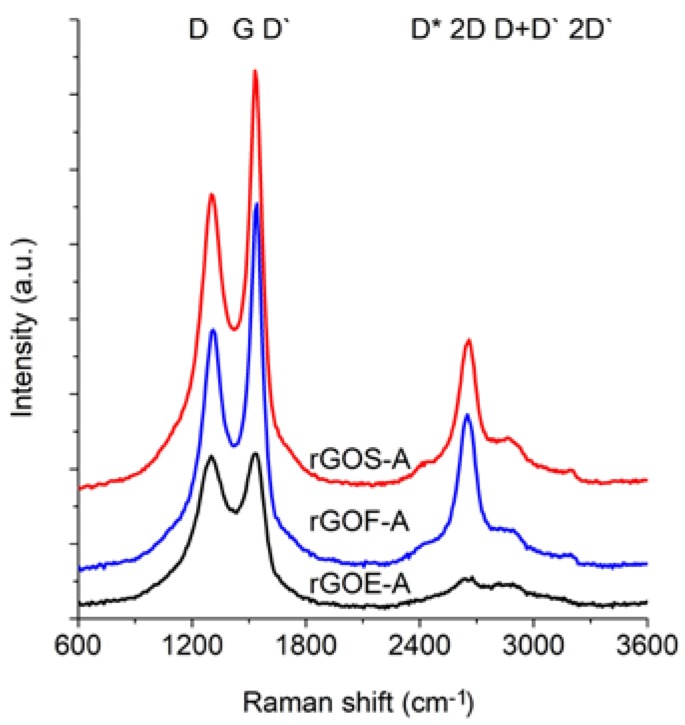
Raman spectra of the reduced graphene oxides obtained by thermal reduction of graphite oxide (oxidation method A).

**Figure 12:**
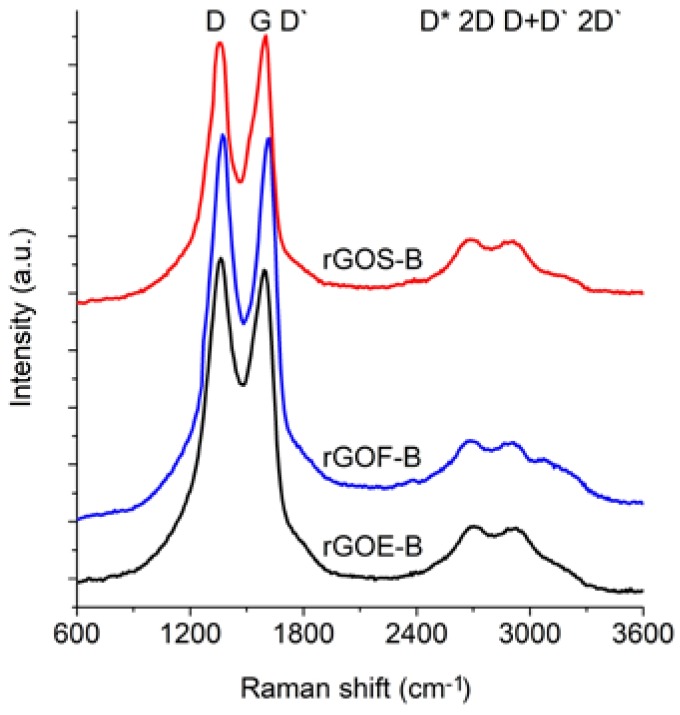
Raman spectra of the reduced graphene oxides obtained by thermal reduction of graphite oxide (oxidation method B).

**Figure 13:**
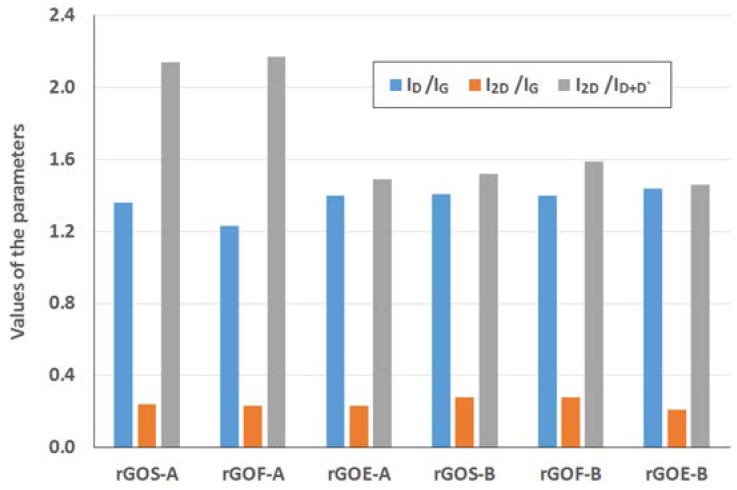
Average values of the parameters I_D_/I_G_, I_2D_/I_G_, and I_2D_/I_D+D’_ obtained from the Raman spectra of the graphite oxides.

The Raman spectra of the reduced graphene oxides obtained by methods A and B are presented in Figure 11 and Figure 12, respectively. The position and the FWHM of the peaks are shown in Table 7 while the average values of the parameters: I_D_/I_G_, I_2D_/I_G_, and I_2D_/I_D+D’_ are shown in Figure 13. These spectra exhibit the same bands as those of the GROs. However, the G bands shift towards lower wavenumbers compared to the G bands in the Raman spectra of the GROs. This difference is associated with the lower content of oxygen groups in the rGOs and the regeneration of the graphite structure. At the same time, the intensity of the D band increases, which indicates the formation of new defects in the structures during the reduction process.

Reduced graphene oxides are made of graphene layers with residual oxygen groups attached to the planes and edges. The obtained materials exhibit intense G band and D bands, which confirms the presence of defects in the graphene layers. The FWHM values of the D and G peaks of the rGOs are smaller than those of the GROs, which indicates the presence of fewer defects in the structure (associated with a lower oxygen content). In the spectra of the rGOs, the intense G band is accompanied by a D’ band, the intensity of which is proportional to the amount of defects in the structure. The FWHM of this band is ~61–68 cm^−1^, which is comparable to the corresponding FWHM values of the GROs (~61–66 cm^−1^), thereby confirming that both the reduction and oxidation process contribute to the formation of defects in the graphene layers.

The rGO-Bs are characterized by D* and 2D bands with larger FWHM values compared to those of the rGO-As, which indicates that these samples have fewer graphene layers in their aggregates (as a reminder: the average number of layers determined using the XRD method is 22–28 for the rGO-As and 8–11 for the rGO-Bs). Simultaneously, the FWHM values obtained for the rGO-Bs are larger than those obtained for the GRO-Bs. This result indicates that the aggregates of the rGOs contain fewer graphene layers than the aggregates of the GROs (also, the intensities of these bands are weaker).

The intensity of the D peak is lower for the rGO-A samples than the rGO-B samples, confirming that these materials contain fewer defects. This feature further results in lower I_D_/I_G_ ratios. The higher I_D_/I_G_ ratios obtained for the rGO-B samples confirm that despite thermal reduction of these structures, more defects and imperfections are present in these samples than in the rGO-A structures. This difference can be explained by the higher oxygen content in the rGO-Bs, which is related to larger distances between the layers and a greater degree of exfoliation. The I_D_/I_G_ values determined for the rGOs depend on the type of graphite used and increase in the order: flake (F) < scale (S) < synthetic (E) graphite (the lowest I_D_/I_G_ values are obtained for the rGOs prepared from flake graphite, which indicates that these samples contain the fewest defects). This trend can be related to the size of the graphene layers (L_a_) determined using the XRD method, in which the opposite trend is observed (synthetic < scale < flake graphite). Based on the measurements performed, smaller graphite crystallites lead to larger amounts of defects in the obtained rGO structures. This relationship was also confirmed in the papers [1,37], where the authors determined that graphene oxides containing the fewest defects were obtained from graphite precursors with the largest crystallites.

Analysis of the Raman spectra of the rGOs shows that the intensity of the 2D band is much lower than that of the G band. The I_2D_/I_G_ ratio is related to the number of graphene layers, and larger values of this ratio are observed for the rGOs than for the corresponding GROs. The smallest values are obtained for rGOE-B, which indicates that this material has the fewest layers in its aggregates. Due to the type of graphite used, the I_2D_/I_D+D’_ ratio is the smallest for the GRO-Es. Please note that these materials are characterized by the smallest quantity of sp^2^ hybridized carbon and the largest quantity of sp^3^ hybridized carbon (determined by XPS measurements), indicating a high degree of destruction of the graphene layers. Furthermore, the I_2D_/I_D+D’_ ratios are the smallest in this case. Conversely, the rGO-Fs are characterized by the largest quantity of sp^2^ hybridized carbon and the smallest quantity of sp^3^ hybridized carbon, which indicates the presence of fewer defects in these graphene structures.

## 4. Discussion

From comparison of the different oxidation methods, method B is determined to be more effective for introducing oxygen into the graphite structure (more carbonyl and quinone groups and fewer carboxyl groups are present). Furthermore, comparison of the type of precursor (graphite) used shows that the synthetic graphite (E) is the least susceptible to oxidation. This conclusion is confirmed by the results of RS. The largest I_D_/I_G_ ratio and the smallest I_2D_/I_G_ and I_2D_/I_D+D’_ ratios are obtained for the oxides prepared from synthetic graphite (E), which indicates that this graphite is the most susceptible to oxidation and exfoliation. In the case of the oxides obtained from flake graphite (F), the smallest I_D_/I_G_ ratio and the largest I_2D_/I_G_ and I_2D_/I_D+D’_ ratios are obtained. Based on the Raman spectra, an increase in the D band is observed for the GROs in comparison to that of the unoxidized graphite. This increase is related to the formation of sp^3^ hybridized bonds as a result of the oxidation of graphite. The presence of oxygen atoms causes both an increase in the interplanar distance and changes in the characteristics of the vibrations of the material’s lattice.

Please note that rapid degradation of the oxygen groups occurs during thermal exfoliation and reduction of GRO. This degradation is accompanied by an increase in pressure due to the evolution of CO_2_, CO and H_2_O, which leads to a weakening of the van der Waals forces between graphene planes, and as a consequence, individual graphene sheets are obtained. In such a process, complete elimination of oxygen from the graphite structure cannot be achieved, and the obtained product may contain even several percent of oxygen depending on the degree of reduction.

Analysis of the data obtained from XPS measurements reveals that the degree of reduction (represented as the C/O ratio) changes in the order rGOE < rGOS < rGOF.

The I_D_/I_G_ values, which indicate the content of defects in the structure, of the rGOs increase in the order rGOF < rGOG < rGOE. This trend is related to the size of the graphene layers (L_a_), which exhibits the opposite trend (rGOE < rGOS < rGOF).

As we have proven, both the type of graphite used and the method of oxidation have a fundamental influence on the properties of the resulting rGOs. Further research on the prepared materials will include studies of their electrical properties. The sensitivity of selected parameters to the surrounding environment will also be tested (the rGOs will be applied as sensor layers in gas sensing devices).

## 5. Conclusions

This work involved various advanced research methods that enable deeper analysis of the changes related to the incorporation of oxygen atoms into graphite oxides and reduced graphene oxides.

The research facilitated the development of preliminary criteria for the selection of a graphite precursor for the preparation of reduced graphene oxides with the desired textural and structural parameters. The reduced graphene oxides obtained from flake graphites, which had the largest crystallite diameter among the analysed graphites, were characterized by the highest degree of reduction and the fewest defects. Moreover, in each investigated case, large crystallite dimensions in the graphite precursor corresponded to large crystallite dimensions in the obtained reduced graphene oxide (regardless of the oxidation method).

This work also showed that the oxidation method has a large influence on the studied parameters of the obtained materials. The research allowed to indicate which of three graphite precursors is the most susceptible to oxidation and what structural and textural parameters will graphene materials have after exfoliation/reduction process. Graphite oxides obtained by method B (modified Tour’s method) were characterized by higher content of oxygen groups in comparison to method A (Hummers’ method). The flake graphite, characterized by the largest diameter of crystallites, proved to be the most suitable material for the preparation of graphene materials. Reduced graphene oxides obtained from flake graphite are characterized by a greater reduction degree and less amounts of defect (compared to reduced graphene oxides obtained from scale and synthetic graphite).

## Figures and Tables

**Table 1:** Conditions of graphite oxide preparation.

Symbol	Condition of Preparation
Method A	graphite (1 g); H_2_SO_4_ (30 mL); NaNO_3_ (3 g); KMnO_4_ (3 g); 2 h
Method B	graphite (1 g); H_2_SO_4_ (45 mL); H_3_PO_4_ (5 mL); KNO_3_ (1.5 g); KMnO_4_ (5 g); 5 h

**Table 2:** The structural parameters of the graphites determined using the XRD method, nm.

Graphite	L_a_	L_c_	d_002_
scale (S)	59	29	0.3379
flake (F)	82	20	0.3362
synthetic (E)	50	26	0.3369

**Table 3:** FWHM values and position of the D, G and 2D bands in the Raman spectra.

Graphite	D Band	G Band	2D Band	A_G_/A_D_
	FWHM/Position of the Band (cm^−1^)			
scale (S)	52/1346	16/1573	67/2695	7.44
flake (F)	43/1344	19/1569	102/2685	7.55
synthetic (E)	38/1347	17/1576	65/2699	8.36

**Table 4:** The structural parameters of the graphite oxides determined using the XRD method, nm.

	d_001_	L_a_	L_c_
**GROS-A**	0.7795	32	4
**GROF-A**	0.7926	31	5
**GROE-A**	0.7687	23	5
**GROS-B**	0.7983	30	4
**GROF-B**	0.8040	30	3
**GROE-B**	0.7939	25	4

**Table 5:** FWHM values and position of the bands in the Raman spectra of the graphite oxides.

	G Band	D Band	D’ Band	D* Band	2D Band	D+D’ Band	2D’ Band
	FWHM/Position of the Band (cm^−1^)						
**GROS-A**	114/1563	214/1361	64/1609	133/2558	226/2700	251/2905	130/3118
**GROF-A**	130/1563	223/1360	65/1607	150/2524	229/2697	274/2910	124/3105
**GROE-A**	125/1553	219/1350	66/1598	110/2546	237/2692	256/2901	99/3094
**GROS-B**	120/1566	217/1364	61/1606	131/2556	229/2703	247/2901	126/3114
**GROF-B**	133/1568	226/1363	62/1603	148/2522	232/2700	270/2906	120/3101
**GROE-B**	128/1556	222/1353	63/1595	108/2544	240/2695	252/2897	95/3090

**Table 6:** The structural parameters of the reduced graphene oxides determined using the XRD method, nm.

	d_00X_	d_002_	L_c_	L_a_	N
**rGOS-A**	0.4031	0.3393	8	21	25
**rGOF-A**	0.4040	0.3383	9	25	28
**rGOE-A**	0.3981	0.3373	7	18	22
**rGOS-B**	0.4072	0.3421	4	10	11
**rGOF-B**	0.4151	0.3421	4	11	11
**rGOE-B**	0.4055	0.3440	3	8	8

**Table 7:** FWHM values and position of the bands in the Raman spectra of the reduced graphite oxides.

	G Band	D Band	D’ Band	D* Band	2D Band	D+D’ Band	2D’ Band
	FWHM/Position of the Band (cm^−1^)						
**rGOS-A**	109/1533	198/1349	62/1598	132/2511	202/2646	232/2825	162/3048
**rGOF-A**	125/1543	217/1353	65/1600	156/2526	214/2657	251/2850	174/3065
**rGOE-A**	119/1550	215/1358	68/1607	143/2540	250/2666	256/2867	165/3098
**rGOS-B**	118/1546	198/1334	61/1594	151/2516	239/2622	244/2801	156/3014
**rGOF-B**	122/1558	211/1349	62/1599	178/2527	242/2645	258/2816	150/3031
**rGOE-B**	120/1550	202/1353	63/1607	168/2540	255/2665	256/2837	140/3040
